# Surgical, Ultrasound Guided Drainage, and Medical Management of Tuboovarian Abscesses

**DOI:** 10.1155/2014/501729

**Published:** 2014-03-04

**Authors:** Frank A. Crespo, Dervi Ganesh, Kaming Lo, Kevin Chin, Paul Norris, Nahida Chakhtoura

**Affiliations:** 1 Department of Obstetrics & Gynecology, Jackson Memorial Hospital, University of Miami Miller School of Medicine, Miami, FL, USA; 2 Holtz Children’s Hospital, Suite 3062, 1611 NW 12th Avenue, Miami, FL 33136, USA

## Abstract

**Objective.:**

To compare surgical, ultrasound guided drainage, and medical management of tuboovarian abscesses (TOA) and determine if different characteristics in patient presentation influence treatment and outcome.

**Methods.:**

Retrospective cohort study of 158 patients admitted to Jackson Memorial Hospital between 2007 and 2012 with a TOA.

**Results.:**

Patients treated with IV antibiotics (IV) alone were hospitalized for 5.59 days (SD 2.52), IV antibiotics and US guided drainage (IV/US) were hospitalized for 9.63 days (SD 7.58), and IV antibiotics and surgery (IV/surgery) were hospitalized for 8.14 days ((SD3.9), (*P* < 0.001)). A total of 52 patients were readmitted with TOA; 41.8% were treated with IV; 26.9% were readmitted with IV/US; 7.1% were readmitted with IV/surgery (*P* < 0.022). Patients with a TOA measuring 0–8 cm were hospitalized for 5.97 days (SD 4.24), while those greater than 8 cm were hospitalized for 7.71 days ((SD 4.69), (*P* < 0.029)). Patients treated with a triple antibiotic regimen were hospitalized for 8.42 days (SD 5.70) versus 5.8 days (SD 3.24) when receiving an alternative regimen (*P* < 0.002).

**Conclusions.:**

Longer hospitalization in patients treated uniformly with either triple antibiotics, ultrasound guided drainage, or surgery represents a delay in optimal treatment. Tailoring treatment plans based on patient presentation may allow for shorter hospital stays and improved morbidity.

## 1. Introduction

For centuries, pelvic inflammatory disease (PID) has been a leading cause of morbidity and mortality in the field of gynecology. Several outpatient and inpatient costs have been attributed to this ascending polymicrobial infection. In fact, PID accounts for over 200,000 hospitalization and 100,000 surgical procedures annually; over 1 billion dollars is spent on PID as a sole entity, and another 1 billion is spent on the sequelae of PID annually [1]. From ectopic pregnancy, infertility, to the elusive chronic pelvic pain, PID seems to be at the root of major gynecologic disease.

Chronic PID leads to tubal scarring and blockage, which may ultimately result in an anaerobic growth environment where *E. coli*, *Bacteroides fragilis*, and *Peptostreptococcus* may thrive. A delay in treatment of these pathogens or a recurrent history of PID may consequently lead to the formation of tuboovarian abscesses (TOA). Approximately one-third of hospitalized patients with PID have been found to have a TOA. Those at greatest risk include women aged 15–24 who have had multiple sexual partners, a prior history of PID, or who are HIV positive [1–3].

The Centers for Disease Control and Prevention (CDC) has acknowledged PID as a serious health dilemma. Various antibiotic regimens for the treatment of PID in the inpatient and outpatient settings have been reported. However, the management of TOA is less clear. As per CDC recommendations, every patient diagnosed with a TOA should be admitted for 24 hours of observation [4]. During this time, patients may be evaluated for risk of sepsis or rupture. However, no specific antibiotic regimen or treatment plan is recommended, other than a 14-day course of metronidazole and doxycycline upon discharge. Perhaps these vague recommendations may stem from a lack of evidence supporting one treatment over another.

Much debate exists over the ideal antibiotic regimen for TOA. A single agent broad-spectrum antibiotic (i.e., cefoxitin) plus doxycycline has shown a 75% clinical response rate, equivalent to that of a clindamycin and aminoglycoside regimen [5]. However, other studies suggest that a triple antibiotic regimen (i.e., ampicillin, gentamicin, and clindamycin) is superior, with an 85% cure rate [6]. No official recommendation exists, leaving institution and provider preference as the determinant in treatment selection.

Aside from intravenous antibiotics (IV), TOA may also be treated by drainage under ultrasound (US) guidance or laparoscopy, as well as laparotomy (i.e., a fertility sparing unilateral oophorectomy or complete bilateral salpingo-oophorectomy with or without hysterectomy). Approximately 25% of patients with TOA will require surgical management [7]. Larger TOA have been shown to respond better to surgical management. 35% of abscesses measuring 7–9 cm and 60% measuring ≥10 cm ultimately require surgical management; abscesses >8 cm more often require drainage or surgery and are associated with longer hospitalization [5, 8]. Moreover, ultrasound guided drainage of TOA with concomitant use of intravenous antibiotics is a safe and effective alternative; compared to IV antibiotics alone, ultrasound guided drainage may lead to shorter hospitalizations and more rapid resolution of symptoms [9].

Prior to choosing a treatment modality, careful patient selection should be performed. However, no study has compared all treatment modalities and offered an algorithm for patient selection. The purpose of this study was to compare surgical, ultrasound guided drainage, and medical management of TOA. We hypothesized that different characteristics in patient presentation, for example, comorbidities, immune status, and tuboovarian abscess size, influence ultimate treatment and outcome. Duration of hospitalization was hypothesized to be different between treatment modalities. This study aims to clarify the management of TOA and offer a potential algorithm for clinicians to follow.

## 2 Materials and Methods

Institutional review board approval by Jackson Memorial Hospital’s Human Subjects Research Office was obtained before commencement of our retrospective cohort study. Inclusion criteria consisted of admission to Jackson Memorial Hospital with a diagnosis of TOA via ICD9 code of 614.2 along with diagnostic features of TOA on imaging. Imaging consisted of transvaginal ultrasonography and/or computed tomography. Descriptive features on imaging included complex, multilocular cystic masses with thick, irregular walls, partitions, and internal echoes. Patients were excluded if their age was less than 18, if they had an admitting diagnosis solely of PID via ICD code of 614.9, and if they had an associated ectopic or intrauterine pregnancy.

227 charts were reviewed from June 6, 2007, to December 12, 2012. After exclusion criteria, 158 patient charts were included in the final analyses. Charts were then randomly assigned and reviewed by three trained investigators. Data was abstracted for patient demographics, clinical presentation, diagnostic TOA features, abscess size, treatment modality, and hospital course. All patient identifiers were removed from the final database.

The outcome measures of this study included duration of hospitalization, readmission, and chronic pelvic pain. The patient’s initial date of admission was determined to be day one of hospitalization; conversely, date of discharge was considered the final day of hospitalization. The duration of hospitalization was estimated to the nearest day. Readmission to the hospital was documented if the patient was readmitted with a diagnosis of TOA at any time since discharge. Chronic pelvic pain was documented if the patient had 6 months or greater of pelvic pain after discharge. Using an electronic medical health record, history and physical notes, follow-up clinic notes, ER documentation, and discharge summaries were all reviewed. Charts with missing data or patients lost to follow-up were excluded from the final analyses.

Descriptive statistics were used to report patient demographics such as age, race, HIV status, abscess size, and duration of hospitalization. In order to test the primary hypothesis, one-way analysis of variance was used to assess the overall difference in days hospitalized between treatment modalities. Pairwise comparisons using Bonferroni’s correction evaluated differences in duration of hospitalization and readmission between all three treatment groups. Secondary analyses included comparisons of patient presentations on use of triple antibiotics, HIV status, and TOA size. Two-sample *t*-tests were used for continuous variables and chi-squared or Fisher’s exact tests were used for categorical variables where appropriate. All statistical analyses were performed using SPSS (version 21.0; IBM Corp, Armonk, NY) with a type I error rate of 0.05.

## 3. Results

The mean age of this cohort study was 37.39 years (SD 10.25). 13.29% were HIV positive, 52.53% were black, and 35.44% were Hispanic. 25 patients had a TOA measuring 0–4 cm, and 74 patients had a TOA measuring greater than 7 cm (Table 1). 45 patients had a TOA measuring greater than 8 cm. 10 patients were excluded from statistical analyses secondary to missing TOA size.

All patients in the study were treated with IV antibiotics. A consistent antibiotic regimen was not noted, as additional antibiotics were added after a failed clinical response. The most common regimens included gentamicin plus clindamycin, cefoxitin plus doxycycline, and a triple antibiotic regimen consisting of ampicillin, gentamicin, and clindamycin. Patients treated with a triple antibiotic regimen were noted to have a greater duration of hospitalization (Table 2). Those treated with a triple antibiotic regimen were hospitalized for 8.42 days (SD 5.70) versus 5.8 days (SD 3.24) for those receiving an alternative regimen (*P* < 0.002).

**FIGURE 1:**
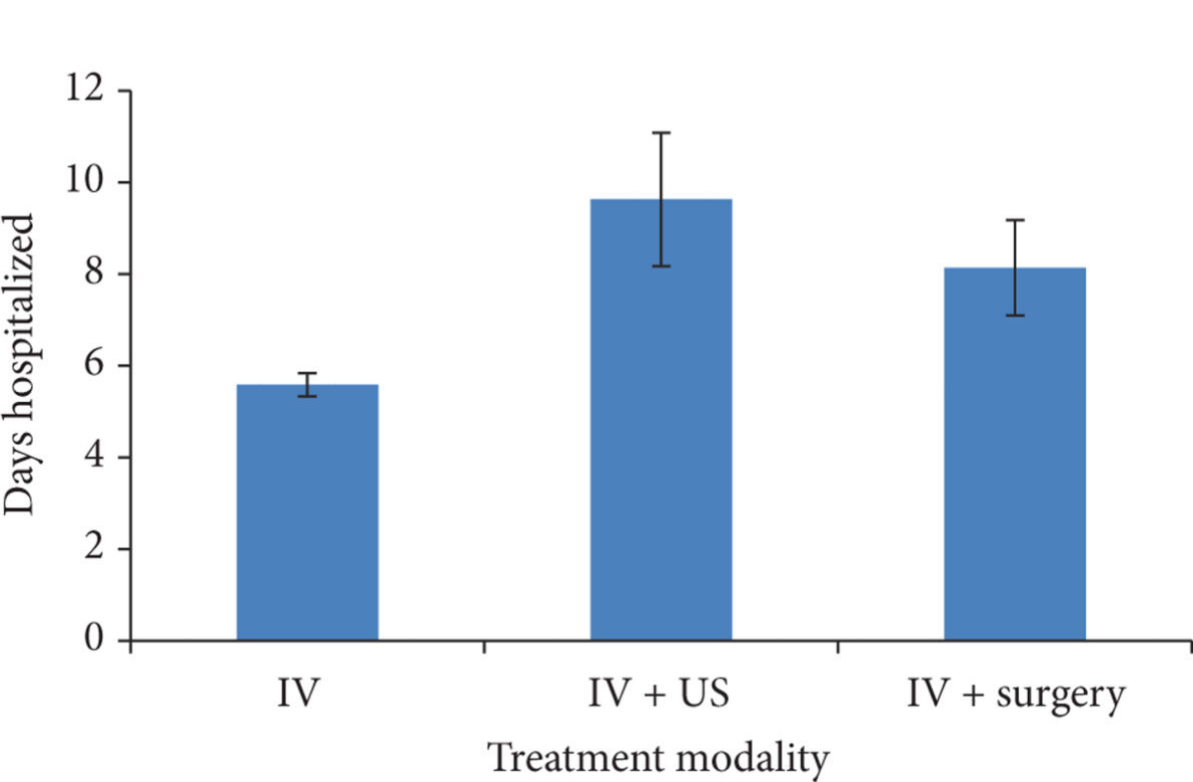
Mean duration of hospitalization in days with standard errors for comparisons between treatment modalities. Duration of hospitalization was different between all groups (*P* < 0.001). Post hoc pairwise comparison using Bonferroni’s correction showed the significant difference for duration of hospitalization between the IV group and the IV/US group (*P* < 0.001).

Compared to patients who were solely treated with IV antibiotics, those who ultimately required ultrasound guided drainage or surgery were hospitalized for the greatest duration of time. Patients treated with IV antibiotics (IV) alone were hospitalized for 5.59 days (SD 2.52). Those treated with both IV antibiotics and US guided drainage (IV/US) were hospitalized for 9.63 days (SD 7.58); and those who were treated with both IV antibiotics and surgery (IV/surgery) were hospitalized for 8.14 days (SD 3.9). One-way analysis of variance revealed an overall significant difference on duration of hospitalization between the three treatment groups (*P* < 0.001). The greatest difference for duration of hospitalization was between the IV group and IV/US group (Figure 1). Post hoc pairwise comparison using Bonferroni’s correction showed a statistically significant difference for duration of hospitalization between these two groups (*P* < 0.001).

**FIGURE 2:**
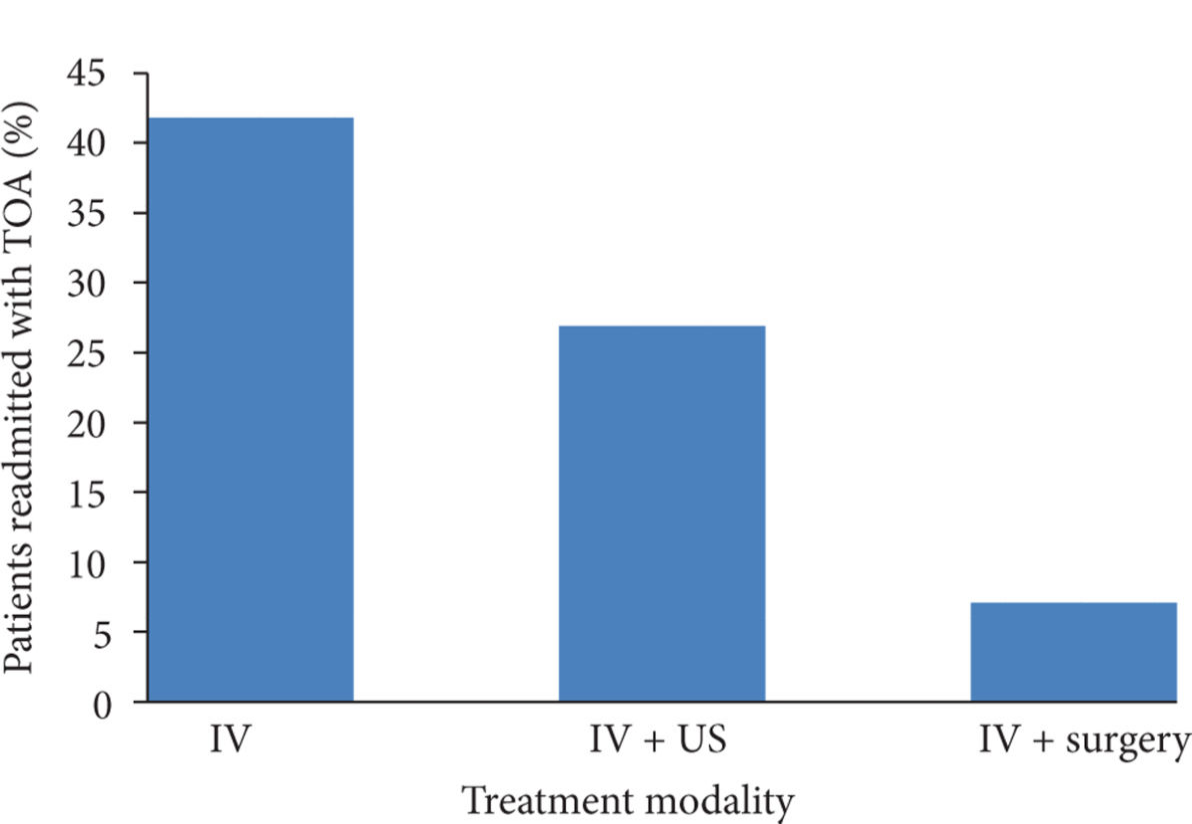
Proportion of readmission between treatment modalities. Readmission with TOA was different between all groups (*P* < 0.022). Post hoc comparison using Bonferroni’s correction showed the significant difference for readmission between the IV group and the IV/surgery group (*P* < 0.048).

Readmission with TOA was different between all treatment groups (Figure 2). A total of 52 patients were readmitted with TOA. 41 patients were treated with IV, 7 were treated with IV/US, and 1 was treated with IV/surgery (Fisher’s exact test, *P* < 0.022). 41.8% of the patients treated with IV antibiotics alone were readmitted. Moreover, 26.9% of patients treated with IV/US and 7.1% of patients treated with IV/surgery were readmitted. Post hoc comparison using Bonferroni’s correction showed the statistically significant difference for readmission between the IV group and the IV/surgery group (*P* < 0.048).

**FIGURE 3:**
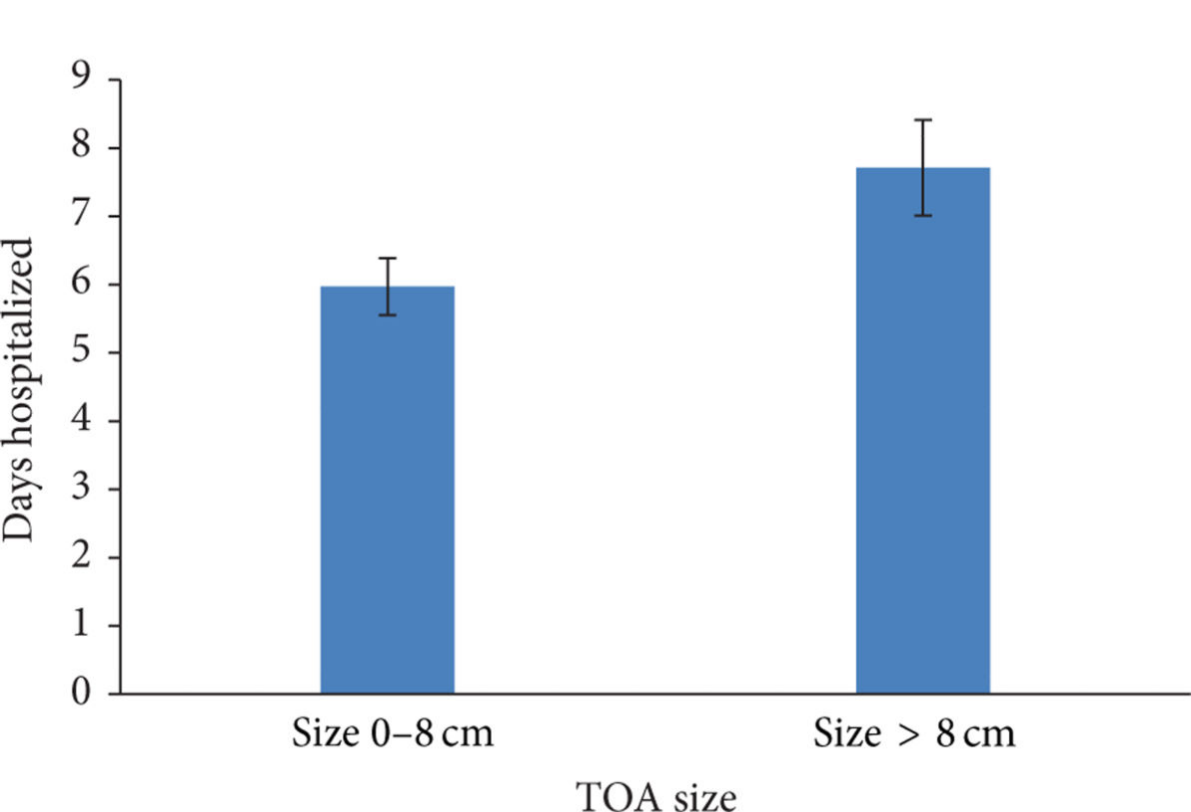
Mean duration of hospitalization in days with standard errors for comparison. TOA of larger size were hospitalized for a greater duration of time. Patients with a TOA measuring 0–8 cm were hospitalized for 5.97 days (SE 0.42), while those greater than 8 cm were hospitalized for 7.71 days (SE 0.7), (*P* < 0.029).

**FIGURE 4:**
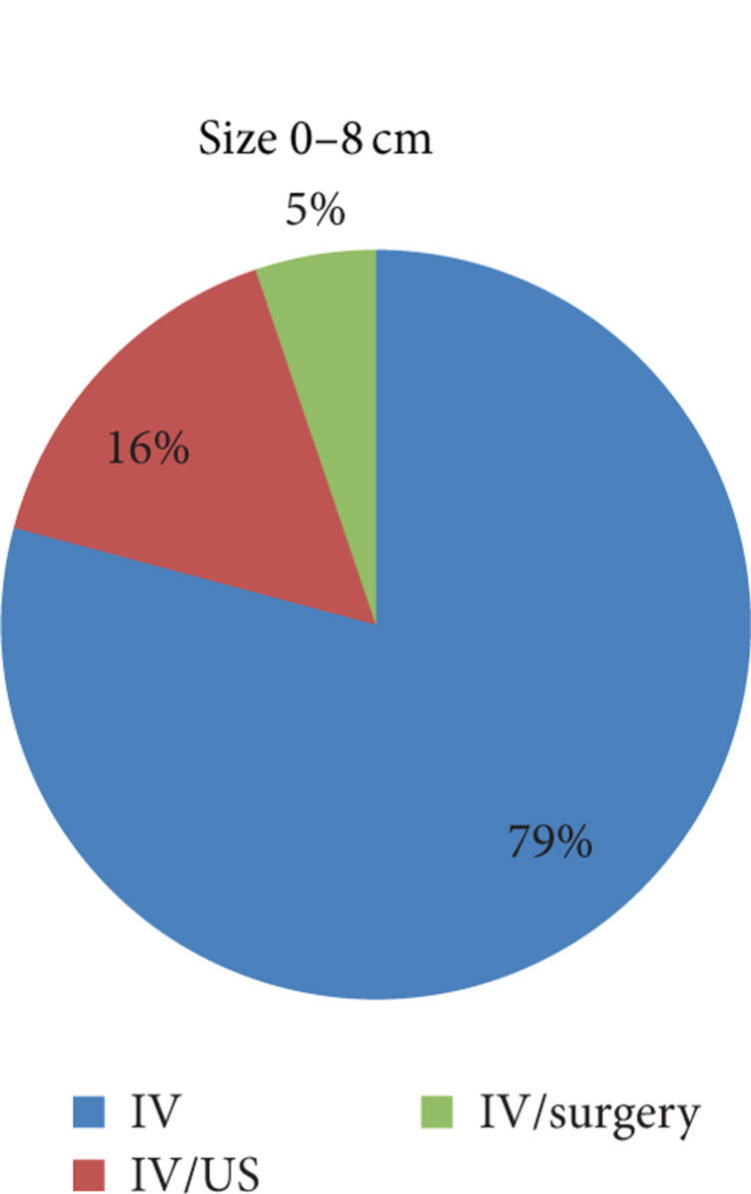
Percentage of each treatment modality used for TOA measuring 0–8 cm.

**FIGURE 5:**
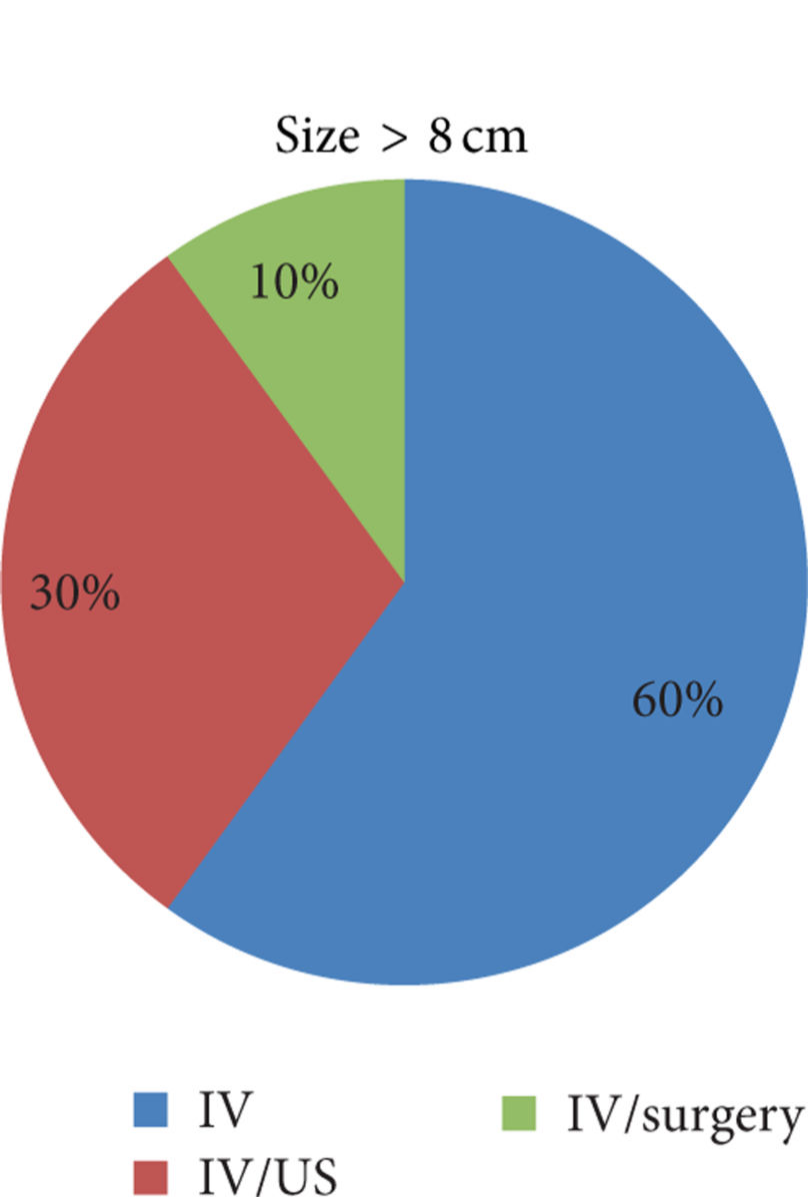
Percentage of each treatment modality used for TOA measuring >8 cm.

TOA of larger size had a greater duration of hospitalization (Figure 3). Patients with a TOA measuring 0–8 cm were hospitalized for 5.97 days (SD 4.24), while those greater than 8 cm were hospitalized for 7.71 days ((SD 4.69), (*P* < 0.029)). There was also a trend towards surgical management for TOA of larger size. Of those patients with a TOA measuring 0–8 cm, 79.17% were treated with IV, 15.63% were treated with IV/US, and 5.2% were treated with IV/surgery. Of those with a TOA measuring greater than 8 cm, 60% were treated IV, 30% were treated with IV/US, and 10% were treated with IV/surgery (Figures 4 and 5).

A total of 31 patients presented with chronic pelvic pain after discharge. There was no statistically significant difference for chronic pelvic pain between all treatment groups (*P* < 0.067).

Moreover, there was no statistically significant difference for duration of hospitalization, readmission, or chronic pelvic pain between HIV positive and non-HIV positive patients. HIV positive patients were hospitalized for 7.39 days (SD 2.09), while non-HIV patients were hospitalized for 6.38 days ((SD 4.66), (*P* < 0.310)).

## 4 Discussion

A clear delineation between TOA size and optimal treatment needs to be made. Few studies have been able to address this point. This study is consistent with previous studies in reporting that larger TOA may require surgical management. We found that TOA greater than 8 cm in size are associated with lengthier hospitalization and increased rates of readmission when managed conservatively.

Antibiotic administration is based on ability to penetrate the abscess cavity. Larger TOA, resulting from chronic untreated PID, may lead to a scarred anaerobic environment resistant to antibiotic penetration. After 9 cm, approximately 60% of abscesses require surgery [1]. However, patients with TOA greater than 8 cm were still treated with IV antibiotics, some for 4–5 days before turning to surgery.

Patients treated with a surgical modality have longer hospital stays compared to IV antibiotics alone [8]. Several theories may exist to explain this phenomenon. Despite evidence that larger TOA may better respond to surgical management, clinicians may hesitate to treat patients surgically. This results in suboptimal treatment and ultimately increased hospital costs. Another explanation is that larger TOA may harbor more virulent pathogens, requiring a longer duration of treatment.

Clinicians may opt to initiate conservative medical management for larger TOA prior to a surgical modality [1]. However, it has been shown that surgical management via ultrasound guided drainage is a safe and efficacious alternative compared to IV antibiotics alone. In one study, patients treated primarily with ultrasound guided drainage had short hospital stays and faster resolution of symptoms [9]. Cost of care may be another reason that optimal treatment is being delayed. Providers may initiate antibiotics in an effort to avoid surgical treatment, as it may appear more costly. However, patients with larger TOA end up staying in the hospital longer, eventually requiring surgery. Providing patients with ultrasound guided drainage or surgery, from the onset, may actually shorten hospital stays and decrease hospital costs.

Patients treated with triple IV antibiotics were also noted to have longer hospitalizations. Again, we believe that there was a possible delay in initiating optimal treatment. 32 patients were initially placed on IV gentamicin and clindamycin. After persistent fever or pain, ampicillin was added to their regimen. Some studies indicate that ampicillin, gentamicin, and clindamycin are superior to a gentamicin and clindamycin regimen [6]. Starting with triple antibiotics may thus provide optimal treatment and decrease hospital stay.

This study also noted that patients treated with IV antibiotics alone were more likely to be readmitted to the hospital with TOA. No other studies have looked at this outcome measure. After noting a clinical response, providers discharged patients after 48 hrs of IV antibiotics. Although IV antibiotics may have quelled patient symptomatology, this was temporary, and cure was not guaranteed. In fact, of the 52 patients who were readmitted with a diagnosis of TOA, 41 were treated with IV antibiotics alone. Some of these patients were readmitted multiple times. Providers failed to appreciate the chronic nature of TOA and the likelihood of recurrence, thus resorting to less optimal treatment. Surgical treatment, especially for larger TOA, may decrease rates of readmission.

In terms of chronic pelvic pain, there was no statistical difference observed between all treatment groups. However, given the long-term follow-up required to assess chronic pelvic pain, we may have not been able to detect this measure accurately. For one, patients may have been lost to follow-up. Secondly, providers may have failed to document pelvic pain in their outpatient clinic notes. Finally, 6 months may have not elapsed since the patient’s date of discharge and time of study.

This is the largest study conducted on the management of TOA. This study provides a novel comparison of IV antibiotics, ultrasound guided drainage, and surgery for the treatment of TOA. One of the strengths of the study includes utilization of readmission to hospital, and chronic pelvic pain as outcome measures. Prior studies have also analyzed duration of hospitalization; however, we were able to compare this measure in relation to each treatment modality. Limitations of the study include its retrospective approach, the small sample size, and attending bias in treatment selection.

**FIGURE 6:**
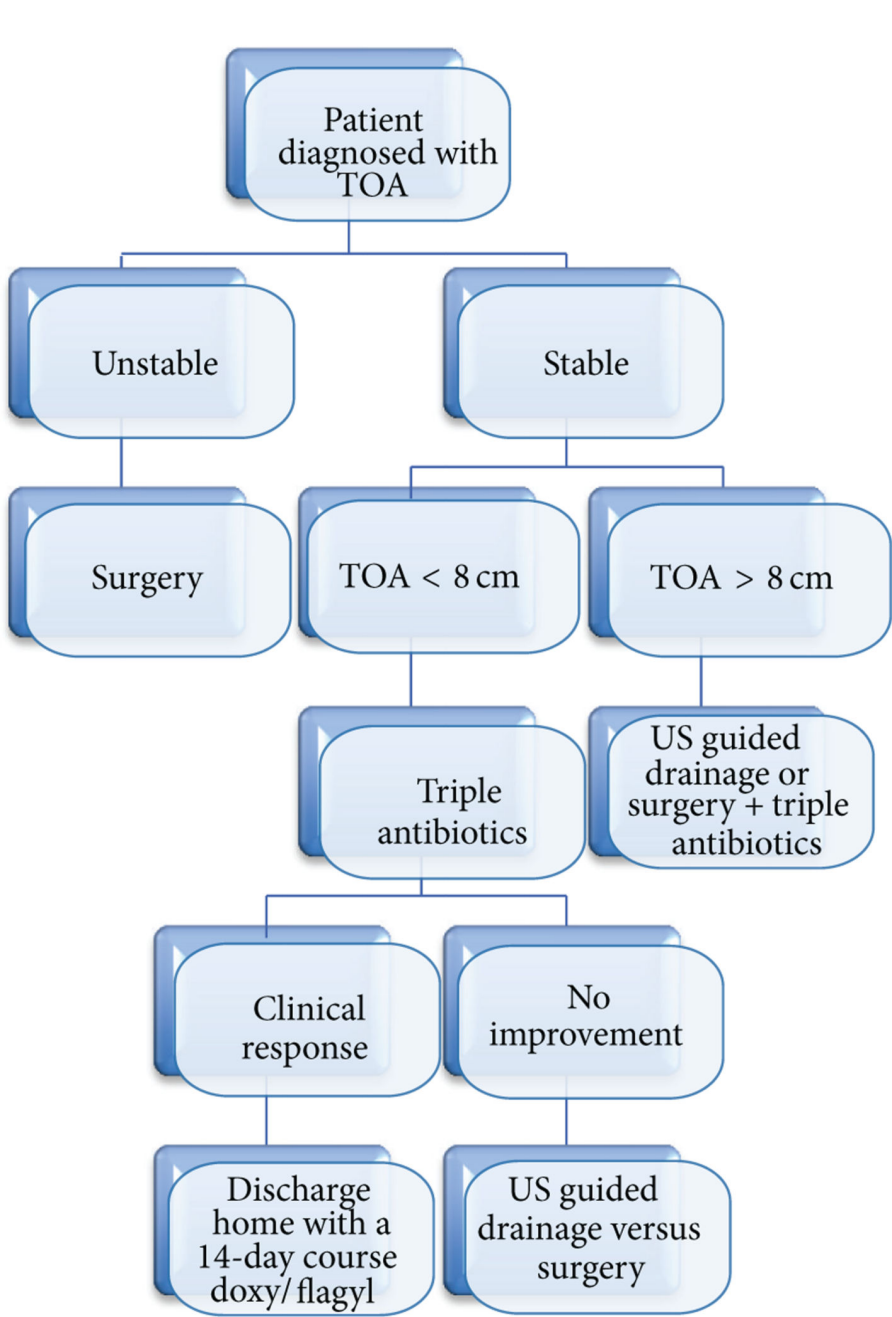
Proposed algorithm for the management of TOA. Patient presents with a TOA. If unstable, proceed with surgical management. If stable, look at TOA size. If size is <8 cm, place patient on triple IV antibiotics for 48 hours. If no clinical response, proceed with US guided drainage or surgery. If there is a clinical response, discharge patient home with 14-day course of metronidazole and doxycycline. If TOA measures >8 cm, proceed with US guided drainage or surgery +/− concomitant IV antibiotics.

Creating a standard algorithm for the treatment of TOA may shorten duration of hospitalization and decrease hospital costs. Based on the study findings, as well as the prior literature on TOA, we proposed such an algorithm (Figure 6).

First, a patient presents with the diagnosis of TOA. If the patient is unstable, the clinician proceeds with surgical management. If stable, TOA size is the next determinant in treatment modality. If the TOA measures <8 cm, the patient will be placed on triple IV antibiotics for 48 hours. If there is no clinical response, the clinician will proceed with ultrasound guided drainage or surgery. If there is a clinical response, the patient will be discharged home with a 14-day course of metronidazole and doxycycline. If the TOA measures >8 cm, the clinician will proceed with ultrasound guided drainage or surgery +/− concomitant IV antibiotics. Implementation of this algorithm may allow for standardization of care. We hope to analyze the effectiveness of this algorithm prospectively.

The findings of this study allow us to identify which patient populations benefit most from either medical, surgical, or ultrasound guided drainage of TOA. Tailoring treatment plans based on patient presentation will potentially allow for shorter hospital stays, decreased rates of readmission, and improved morbidity.

## Figures and Tables

**TABLE 1:** Patient demographics.

	Mean/counts	SD/%
Age	37.39	10.252
Race		
White	19	12
Black	83	52.5
Hispanic	56	35.4
HIV		
Yes	21	13.3
Size		
0–4 cm	25	16.9
4.1–7 cm	49	33.1
>7 cm	74	50

**TABLE 2:** Triple antibiotics versus alternative regimen.

	Triple antibiotics		Other regimens	
	Mean/counts	SD/%	Mean/counts	SD/%
Days hospitalized	8.421*	5.696	5.8	3.237
Pain (CPP)	15	41.7	13	23.2
Readmission	22	43.1	26	32.5

* *P* < 0.002.
